# Genotypic Characterization of Carbapenem-Resistant *Klebsiella pneumoniae* Isolated from an Egyptian University Hospital

**DOI:** 10.3390/pathogens12010121

**Published:** 2023-01-11

**Authors:** Marwa S. Taha, Maha M. Hagras, Marwa M. Shalaby, Yosra Abdelmonem Zamzam, Reham M. Elkolaly, Marwa A. Abdelwahab, Sara Youssef Maxwell

**Affiliations:** 1 Department of Medical Microbiology and Immunology, Faculty of Medicine, Tanta University, Tanta 31527, Egypt; 2 Department of Clinical Pathology, Faculty of Medicine, Tanta University, Tanta 31527, Egypt; 3 Department of Chest Diseases, Faculty of Medicine, Tanta University, Tanta 31527, Egypt

**Keywords:** *Klebsiella pneumoniae*, carbapenem resistance, capsular serotypes, *bla_OXA-48_*, *bla_VIM_*, *bla_KPC_*, *bla_NDM_*, *bla_IMP_*

## Abstract

Globally, *Klebsiella pneumoniae* (*K. pneumoniae*) has been identified as a serious source of infections. The objectives of our study were to investigate the prevalence of multidrug-resistant (MDR) *K. pneumoniae* in Tanta University Hospitals, Gharbia Governorate, Egypt; characterize their carbapenem resistance profiles; and identify their different capsular serotypes. We identified and isolated 160 (32%) *K. pneumoniae* from 500 different clinical samples, performed antimicrobial susceptibility testing, and then used multiplex PCR to detect carbapenemase genes and capsular serotypes K1, K2, K3, K5, K20, K54, and K57. We detected phenotypic carbapenem resistance in 31.3% (50/160) of the isolates; however, molecular assays revealed that 38.75% (62/160) of isolates were carrying carbapenemase-encoding genes. Generally, *bla*_OXA-48_ was the prevalent gene (15.5%), followed by *bla_VIM_* (15%), *bla_IMP_* (7.5%), *bla_KPC_* (4%), and *bla_NDM_* (3.8%). *Bla_VIM_* and *bla_OXA-48_* correlated with phenotypic resistance in 91.67% and 88% of the isolates that harbored them, respectively. Capsular typing showed that the most prevalent pathotype was K1 (30.6%), followed by K57 (24.2%), K54 (19.35%), K20 (9.67%), and K2 (6.45%). A critical risk to community health is posed by the high incidence of multidrug-resistant (MDR) virulent *K. pneumoniae* isolates from our hospital, and our study examines this pathogen’s public health and epidemiological risks.

## 1. Introduction

One of the biggest pressures on healthcare systems around the world is the rising prevalence of antibiotics-resistant clinical bacterial isolates [1,2]. Understanding the genetic factors of antibiotic resistance is essential to stop the spread of MDR bacteria [3]. 

Among these MDR bacteria, *K. pneumoniae* is regarded as one of the top six factors contributing to healthcare-associated infections and drug resistance [4]. As an opportunistic pathogen, *K. pneumoniae* consists of Gram-negative bacilli and is a member of the enterobacterales family that primarily affects people who are immunocompromised or are admitted to hospitals. Numerous ailments, such as sepsis, bacteremia, pneumonia, and urinary tract infections, are attributed to *K. pneumoniae* [5]. 

A sizeable portion of illnesses brought on by *Klebsiella* spp. is a result of two significant pathotypes, notably the MDR and hypervirulent (hv), which eventually produce convergent genetic copies, termed multidrug-resistant and hypervirulent (MDRhv) *Klebsiella* spp. [6]. 

New antimicrobial-resistance genes were initially found in *K. pneumoniae*, and they later spread to further pathogens: carbapenem-resistant *K. pneumoniae* (CRKP) genes (*bla_KPC_*, *bla_OXA-48_* and *bla_NDM-1_*) are examples [7]. The essential pathogenic component, known as the capsule, an extracellular polysaccharide structure that hinders the host immune response and shields the invading pathogens from phagocytosis, is responsible for the increasing death and morbidity rates linked to *K. pneumoniae* infections [8].

*Klebsiella* has at least 79 different capsular varieties, with each depicting the capsular polysaccharide’s (CPS; the K antigen) molecular structure differently. These types have been connected to the severity of the sickness and the type of infection [9]. Several capsular (K) types, mainly K1, K2, K5, K20, K54, and K57, are correlated to invasive septicemia obtained in the community, pneumonia, and liver abscesses [10]. Furthermore, K3 is attributed to rhinoscleroma [11].

Information about capsule serotypes can be quickly retrieved from whole-genome sequence (WGS) data by typing the relevant capsule (K) biosynthesis loci [12]. A chromosomal region of 10–30 kbp and 10–30 genes make up the K locus. The preserved genes for the export and synthesis of capsules are found in the 5′-(galF, cpsACP, wzi, wza, wzb, wzc) and 3′-(ugd) most areas, and they surround the genes that code for the synthesis of capsule sugar, namely Wzy repeat-unit polymerase and Wzx capsule-specific flippase [13].

Molecular capsular typing is the main technique used to categorize *K. pneumoniae* isolates, and it has outstanding consistency and can distinguish between clinical isolates [14]. Multiplex PCRs have been successfully used to identify the capsule repeat-unit polymerase Wzy genes [15]. 

Few studies on MDR *K. pneumonia* capsular typing have been conducted in Egypt [16,17]. Consequently, we assessed the prevalence of nosocomial MDR *K. pneumoniae* infections in our tertiary care hospitals and characterized their carbapenem resistance profiles.

## 2. Materials and Methods

### 2.1. Study Design

We carried out our cross-sectional study in the Tanta University Hospitals’ Clinical Pathology and Medical Microbiology and Immunology Department over the course of a year, from June 2021 to June 2022. The hospitals have a combined capacity of 2040 beds, including 130 ICU beds, and serve over 190,000 patients annually. Our study received permission from Tanta University’s Institutional Review Board for the Faculty of Medicine in Egypt (Approval code 35789/9/22). 

### 2.2. Study Subjects

A total of 500 patients from Tanta University hospital’s Pediatrics, Chest, Medicine, and Intensive Care Unit (ICU) departments were enrolled in this study. The included patients had hospital-acquired infections (HAIS). We studied 160 clinical isolates of *Klebsiella* from 500 samples from different body sites (blood, CSF, urine, wound, and sputum) of 500 patients. 

### 2.3. Identification of Bacterial Isolates 

We gathered blood, CSF, urine, wounds, and sputum samples from different infection sites and quickly sent them to the Microbiology Department laboratory for additional processing. First, we codified the samples, and then we cultivated aerobically at 37 °C on blood agar, nutrient agar, chocolate agar, and MacConkey agar plates (Oxoid, UK) for 24–48 h. We predominantly used routine microbiological methods for the phenotypic detection of isolated pathogens [18]. Thereafter, we further processed only *K. pneumonia*. We verified *K. pneumonia* using the Vitek-2 automated system (Biomérieux, Marcy-LÉtoile, Paris, France) in accordance with the manufacturer’s recommendations. We kept all *K. pneumoniae* isolates at −80 °C in brain–heart infusion broth (20% glycerol; Oxoid, UK) until they were needed.

### 2.4. Antimicrobial Susceptibility Testing and Phenotypic Detection of Carbapenemases 

We performed the modified Kirby–Bauer disc diffusion method to assess the antibiotic susceptibility of all identified *K. pneumoniae* isolates on Muller–Hinton agar (Oxoid, UK) plates. The antibiotics used were amoxicillin/ clavulanic acid (AMO) 20/10 μg, ciprofloxacin (CIP) 5 μg, cefuroxime (CXM) 30 μg, piperacillin–tazobactam (TPZ) 110 μg, cefoxitin (FOX) 30 μg, cefipime (FEP) 30 μg, ceftriaxone (CRO) 30 μg, ceftazidime (CAZ) 30 μg, cefotaxime (CTX) 30 μg, trimethoprim–sulfamethoxazole (SXT) 25 µg, imipenem (IMI) 10 μg, ertapenem (ERT) 10 μg, and meropenem (MEM) 10 μg (Oxoid, UK). We used the modified Hodge test (MHT) to check for carbapenemase production in isolates, which showed intermediate or resistant zones for ertapenem according to CLSI guidelines [19]. We used *E. coli* ATCC 25922 as a susceptible strain and *K. pneumoniae* ATCC BAA-1705 as a positive control. We interpreted data generated by the susceptibility assay using the CLSI 2021 guidelines [19]. The multiple antibiotic resistance (MAR) index of each isolate was estimated according to Tambekar et al.’s method [20].

### 2.5. Multiplex PCR for Capsular Typing of K. pneumoniae and Detection of Carbapenemases-Encoding Genes

We used two distinct multiplex PCR assays to carry out the molecular characterization of the carbapenem resistance genes and capsular typing of *K. pneumoniae*. The K1, K2, K5, K20, K54, K57, and K3 capsular antigens were the targets of the first multiplex PCR typing [21] (Table 1). We utilized primer sets for the carbapenemases-encoding genes *bla_VIM_*, *bla_IMP_*, *bla_KPC_*, *bla_OXA-48_*, and *bla_NDM_* in the second multiplex PCR [22]. (Table 1)

We obtained total genomic DNA using Qiagen DNA extraction kits (Qiagen, Hilden, Germany) in accordance with the manufacturer’s instructions. Then, we kept the extraction at −20 °C until the following stage.

We used Dream Taq TM Green PCR Master Mix (Fermentas, Waltham, MA, USA) to amplify the tested gene as per the manufacturer’s directions using a Bio-Rad PTC-200 Thermal Cycler (Bio-Rad, Hercules, CA, USA). We created the PCR conditions for capsular and carbapenemase genes molecular typing according to Ssekatawa et al.’s method [23]. We electrophoresed PCR products on a 1.5% agarose gel stained with ethidium bromide and photographed with UV illumination. We used a 100-2000 base-pairs standard DNA ladder (Biomatik, Wilmington, DE, USA) for sizing the PCR products.

### 2.6. Statistical Analysis

We analyzed the data with IBM SPSS Statistics for Windows, Version 25.0 (IBM Corp, New York, NY, USA, 2017). We utilized numbers and percentages to present qualitative data. We used a *p*-value of ≤0.05 to determine statistical significance.

## 3. Results

### 3.1. Distribution of Isolated K. pneumoniae in Clinical Samples

We separated *K. pneumoniae* from distinct types of specimens collected from patients admitted at Tanta university tertiary hospital. We collected 500 samples; however, only 160 specimens yielded *K. pneumoniae*, while the remaining specimens either yielded different organisms or provided no growth. Regarding the 160 samples, 80 were isolated from urine, 40 from pus swabs, 20 from sputum, 10 from tracheal aspirates, and 10 from blood (Table 2). 

### 3.2. Antibiotic Susceptibility Patterns and Phenotypic Detection of Carbapenemases 

Based on the disc diffusion assay, the majority of the isolated *K. pneumoniae* showed significant levels of resistance to used antibiotics. Overall, 99.4% of the isolates exhibited resistance to cefotaxime, while 99% showed resistance to amoxicillin–clavulanic acid and ceftazidime. Furthermore, 98.1% of the isolates exhibited resistance to each of cefuroxime and ceftriaxone, whereas 95% and 94.4% were resistant to trimethoprim–sulfamethoxazole and cefepime, respectively. We observed resistance to piperacillin–tazobactam and ciprofloxacin as the next highest among 81.8% of the isolates, followed by cefoxitin (60%). We found the lowest resistance rate corresponding to imipenem and ertapenem (31.3%), followed by meropenem (30%). All carbapenem-resistant isolates (100%) were MHT positive. The MAR index ranged from 0.69 to 1.0.

### 3.3. Carbapenemase-Encoding Genes Distribution

**Figure 1:**
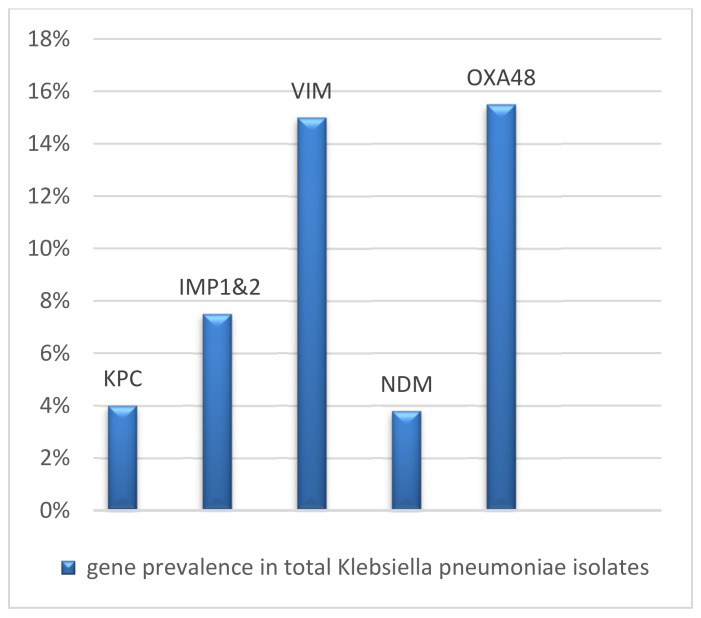
Gene prevalence in *Klebsiella pneumoniae* isolates.

Based on the results obtained by Multiplex PCR assay, out of 160 *K. pneumoniae* isolates, 38.75% (62/160) contained single or mixed carbapenemase genes (Table 3 and Table 4). Of those, *bla_OXA-48_* was the most predominant, with a prevalence of (15.5%) (25/160), followed by *bla_VIM_* (24/160 = 15%), *bla*_IMP_ (12/160 = 7.5%), *bla_KPC_* (7/160 = 4%), and *bla_NDM_* (6/160 = 3.8%) (Figure 1). 

### 3.4. Correlation between Genotypic and Phenotypic Assays

We detected variations between the genotypic and phenotypic resistance of the isolates. A total of 24 isolates harbored the VIM gene, and 22 (91.67%) showed phenotypic carbapenem resistance. This was followed by OXA-48, which showed phenotypic resistance in 22 (88%) of the isolates, then Kpc in 5 (71.43%), IMP-1&2 in 9 (75%), and NDM in 4 (66.67%) (Table 5).

### 3.5. Prevalence of Capsular Types in Isolates Harboring Carbapenemases-Encoding Genes 

**Figure 2:**
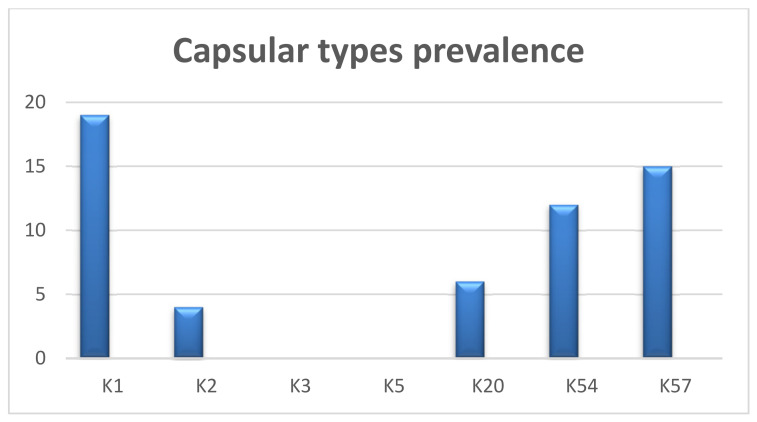
Prevalence of capsular types in carbapenem genotypically resistant isolates.

Our multiplex PCR assay results showed that out of 62 carbapenem-resistant isolates, 19 (30.6%) harbored capsular gene K1, followed by the K57 (15; 24.2%), K54 (12; 19.35%), K20 (6; 9.67%), and K2 genes (4; 6.45%). However, we did not detect the K3 and K5 genes in any of the collected isolates (Figure 2).

### 3.6. Correlation between Source, Antimicrobial Resistance Pattern, Multiple Antibiotic Resistance (MAR) Index, Distribution of Carbapenemase-Encoding Genes, and Capsular Types

The comprehensive correlation between an isolate’s source, antimicrobial resistance pattern, MAR index, carbapenemases genes, and capsular serotypes is displayed in Table 6. We found no significant relations when correlating the different carbapenemase genes detected during our study with capsular serotypes (Table 7).

## 4. Discussion

*K. pneumoniae* has been identified as one of the most popular causes of infections developed in hospitals and the community [24]. The appearance of, MDR and hvKP strains, as well as their rapid clinical propagation, is particularly concerning [25] because their resistance propagation is associated with mobile genetic components, which may additionally hold virulence factors, such as the capsule, siderophores, fimbriae, and lipopolysaccharides (LPS) [26]. Therefore, when highly pathogenic bacteria develop antibiotic resistance, the situation deteriorates [23].

Therefore, we analyzed the frequency of carbapenem-resistant pathogenic *K. pneumoniae* in our tertiary care hospitals to better understand its dangers. Our survey findings show that 50% of *K. pneumoniae* isolates were found in urine, 25% in pus swabs, 20% in sputum, and 6.25% in both blood and tracheal aspirates. Our results are similar to those of a study conducted at Al-Azhar University, Egypt [27]. Additionally, further research carried out in Uganda concluded that most *K. pneumoniae* isolates were obtained from urine, pus, and blood [23].

However, a study in New York conducted by Parrott et al. [28] confirmed that most *K. pneumoniae* isolates were recovered from blood culture, followed by wound swabs. Additionally, Palmeiro et al. [29] found that blood specimens yielded the highest number of isolates. Furthermore, Sedighi P et al. [30] found that throat, urine, and tracheal swabs were the most prevalent samples, while wound, blood, sputum, and abscess cultures showed the least amounts of isolates.

This variation in results may be explained by variations in sample type and case count, sampling conditions, sampling times, sampling locations, sampling countries, and patient general health.

We determined that the isolates we detected in our study were MDR because of their resistance to several types of antibiotics. Meropenem had a 30% resistance rate, whereas imipenem and ertapenem both had a 31.3% resistance rate. This outcome was consistent with the research conducted by Farhadi et al. [31], who observed that 33% of the *K. pneumoniae* isolates were resistant to imipenem. Furthermore, Pereira et al. [32] found that 73 *Klebsiella* isolates found in samples of a urinary tract infection were extremely resistant to IMP.

Moreover, Moghadas et al. [33] found that only 7.5% of their isolates were resistant to IMP, and their survey of North and West Africa highlighted a noticeably increased phenotypic resistance to carbapenems (>50%) [34,35,36,37]. Additionally, a bigger study that examined the South African provinces of Gauteng, KwaZulu-Natal, Western Cape, and Free State found that imipenem, meropenem, and doripenem had overwhelmingly high phenotypic resistance rates of between 47 and 50%, while ertapenem had rates between 84% and 89%.

The disparity in sensitivity patterns between the aforementioned studies may be attributed to various antibiotic policies, the emergence of resistant strains because of indiscriminate antimicrobial therapy, the patient’s immune status, various infection control strategies, or frequent hospitalization.

We must determine whether the *K. pneumoniae* isolate produces carbapenemase in order to conduct epidemiological research and choose the best course of treatment for infections [38]. Regarding the PCR-based carbapenemase gene identification, *bla*_OXA-48_ was the most prevalent, with a genotypic frequency of (15.5%), followed by *bla*_VIM_ type (15%), *bla*_IMP_ (7.5%), *bla*_KPC_ (4%), and *bla*_NDM_ (3.8%). Our findings were consistent with another Egyptian study conducted by Raheel et al. [39], who demonstrated that the *bla_OXA-48_* gene (96.2%) was the most frequently present gene, while the *bla*_KPC_ gene (7.5%) was the least common. Additionally, our result is consistent with recent research that identified the OXA-48 gene and its variations as the most popular gene [35,40,41,42].

OXA- 48 was initially discovered in a *K. pneumoniae* strain from Turkey in 2003. OXA-48 intermittently reached neighboring nations in the southern and eastern Mediterranean Sea, as well as North Africa [43]. This explains why OXA-48 is more common in Tunisia and Egypt than anywhere else [35,41].

Nevertheless, Lopes et al. and Hussein et al. [44,45] found that carbapenem-resistant *K. pneumoniae* isolates had a higher level of *bla_KPC_* expression. Furthermore, El-Monir et al. [46] reported that both *bla*_VIM_ and *bla*_NDM-1_ were the most prevalent genes detected in Egypt. Additionally, further studies showed that the most abundant genes in East Africa were VIM and IMP [40,47], whereas NDM was the most common in South Africa [47,48,49,50].

We recovered more than one resistance gene in 12 *K. pneumoniae* isolates, which is in accordance with many previously published studies that demonstrated that *A. baumannii* and *K. pneumoniae* carry several genes, increasing their likelihood of being multi- or pan-drug resistant [49,51,52,53,54]. However, this can be contested because of the possibility of resistance spreading and the restricted accessibility of antibiotics useful for therapy, as well as the diminishing effectiveness of older antibiotics, such as colistin [55,56].

Our study found that genotypic resistance was generally higher than overall phenotypic resistance. For example, 25 isolates harbored the OXA-48 gene, and 22 (88%) of them showed phenotypic carbapenem resistance. This can be explained by many reports that described OXA-48 and its variant genes’ oxacillinases as having limited hydrolyzing activity for carbapenems [43,57,58]. 

The capsule is a key element affecting *K. pneumoniae*’s pathogenicity. Numerous investigations revealed that the virulence of infections generated by *K. pneumoniae* is influenced by the capsular forms [59,60]. In several strains of *Klebsiella* spp., the gene cluster architecture responsible for producing capsular polysaccharide (CPS) has been previously analyzed [61]. The Wzy and Wzx genes, which generate the proteins necessary for the polymerization and assembly of the various CPS subunits, are situated in a variable region in the center of the CPS locus. As a result, the foundation of PCR capsular typing assays is the significant sequence diversity of the Wzy gene among the various capsular types [62]. Considering this, we identified and characterized the *K. pneumoniae* capsular serotypes that were most clinically relevant using the Wzy gene. 

Our results revealed that (30.6%) of *K. pneumoniae* isolates harbored capsular gene K1, followed by the K57 (24.2%), K54 (19.35%), K20 (9.67%), and K2 genes (6.45%); however, we did not detect the K3 and K5 genes in the collected isolates.

Ssekatawa et al. [23] found that K1, K2, K3, K5, and K20 made up 46.7% of the *K. pneumoniae* isolates; according to capsular typing by heptaplex PCR, while none of the isolates had K54 or K57.

These findings correspond to research conducted by Fung et al. and Chuang et al. [60,63], who concluded that the greatest virulent capsular forms of *K. pneumoniae* K1 and K2 were responsible for septicemia and liver abscesses. Furthermore, according to two surveys conducted in Taiwan by Fang et al. and Lin et al. [59,62], the K1, K2, K3, K5, and K20 genes were the most common capsular types in pneumonic and liver abscess patients. Moreover, Paczosa and Mecsas [64] reported that among the 519 invasive strains they investigated, K2 isolates were found in the largest numbers. In addition, Choi et al. [65] found that K24 was the most prevalent capsule type.

We evaluated the correlation between capsular serotypes and the presence of carbapenemase genes. Our results revealed that carbapenemases genes could not be related to any capsular serotypes (data were statistically not significant). Nonetheless, Soltani et al. [66] found a correlation between *bla*_OXA-48_ and K20 in a study conducted in Iran. 

## 5. Conclusions

Our research highlighted high incidence rates for carbapenem-resistant *K. pneumoniae* in our tertiary care hospital. Although our study did not seek to identify other virulence determinants, the considerable prevalence of carbapenem resistance among capsular serotypes that we found raises the possibility of carbapenem-resistant hypervirulent *K. pneumoniae*, which must be assessed in further studies.

## Figures and Tables

**Table 1:** Primer sequences used in molecular detection of capsular genes and carbapenem resistance genes of *K. pneumoniae* [23].

Primers Targeting Capsular-Encoding Genes		
Target Genes	Primer Sequence (5′-3′)	Amplicon Size (bp)
khe	F: TGA TTG CAT TCG CCA CTG G R: GGT CAA CCC AAC GAT CCT G	428
WzyK1	F: GGT GCT CTT TAC ATC ATT GC R: GCA ATG GCC ATT TGC GTT AG	1283
WzyK2	F: GAC CCG ATA TTC ATA CTT GAC AGA G R: CCT GAA GTA AAA TCG TAA ATA GAT GGC	641
WzxK5	F: TGG TAG TGA TGC TCG CGA R: CCT GAA CCC ACC CCA ATC	280
WzyK20	F: CGG TGC TAC AGT GCA TCA TT R: GTT ATA CGA TGC TCA GTC GC	741
WzxK54	F: CAT TAG CTC AGT GGT TGG CT R: GCT TGA CAA ACA CCA TAG CAG	881
Wzy57	F: CTC AGG GCT AGA AGT GTC AT R: CAC TAA CCC AGA AAG TCG AG	1037
WzyK3	F: TAG GCA ATT GAC TTT AGG TG R: AGT GAA TCA GCC TTC ACC T	549
Primers targeting carbapenemases-encoding genes		
*Bla*_KPC_	F-ATG TCA CTG TAT CGC CGT CT R-TTT TCA GAG CCT TAC TGC CC	538
*Bla*_IMP-1_	F-TGA GCA AGT TAT CTG TAT TC R-TTA GTT GCT TGG TTT TGA TG	139
*Bla*_IMP-2_	F-GGC AGT CGC CCT AAA ACA AA R-TAG TTA CTT GGC TGT GAT GG	139
*Bla*_VIM_	F-GAT GGT GTT TGG TCG CAT A R-CGA ATG CGC AGC ACC AG	390
*Bla*_NDM_	F-GGT TTG GCG ATC TGG TTT TC R-CGG AAT GGC TCA TCA CGA TC	521
*Bla*_OXA-48_	F-TTG GTG GCA TCG ATT ATC GG R-GAG CAC TTC TTT TGT GAT GGC	281

**Table 2:** Prevalence of *Klebsiella pneumoniae* isolated from various clinical specimens.

Sample Type (Number)	*Klebsiella pneumoniae* Isolates
Urine (216)	80 (50%)
Pus swab (103)	40 (25%)
Sputum (78)	20 (12.5%)
Tracheal aspirate (55)	10 (6.25%)
Blood (48)	10 (6.25%)
Total (500)	160 (100%)

**Table 3:** Prevalence of carbapenemase-encoding genes in total *Klebsiella pneumoniae* isolates.

Carbapenemase Gene Tested	Gene Prevalence in Total Klebsiella Pneumoniae Isolates
*Bla*_KPC_	7 (4%)
*Bla*_IMP-1&2_	12 (7.5%)
*Bla*_VIM_	24 (15%)
*Bla*_NDM_	6 (3.8%)
*Bla*_OXA-48_	25 (15.5%)
*Total*	74 (46.25%)

**Table 4:** Distribution of single and mixed carbapenemase genes among the genotypically resistant isolate.

Carbapenemase Gene Tested	Number of Isolates Harboring Carbapenemases
*Bla*_KPC_	4
*Bla*_IMP-1&2_	8
*Bla*_VIM_	21
*Bla*_NDM_	2
*Bla*_OXA-48_	17
*Bla*_NDM_ and *Bla*_OXA-48_	1
*Bla*_KPC_ and *Bla*_IMP-1&2_	1
*Bla*_KPC_ and *Bla*_OXA-48_	1
*Bla*_IMP-1&2_ and *Bla*_OXA-48_	2
*Bla*_VIM_ and *Bla*_OXA-48_	2
*Bla*_VIM_ and *Bla*_NDM_	1
*Bla*_NDM_, *Bla*_KPC,_ and *Bla*_OXA-48_	1
*Bla*_IMP-1&2_, *Bla*_NDM_, and *Bla*_OXA-48_	1
*Total*	62

**Table 5:** Correlation between genotypic and phenotypic resistance.

Carbapenemase-Encoding Genes	Number of Isolates Harboring the Gene	Number of Isolates Harboring the Gene and Phenotypically Resistant	Number of Isolates Harboring the Gene and Phenotypically Sensitive	Percentage of Resistance Conferred by Gene Presence
*Bla_KPC_*	7	5	2	71.43%
*Bla_IMP-1&2_*	12	9	3	75%
*Bla_VIM_*	24	22	2	91.67%
*Bla_NDM_*	6	4	2	66.67%
*Bla_OXA-48_*	25	22	3	88%

**Table 6:** Correlation between source of samples, antimicrobial resistance pattern, MAR index, carbapenemase genes, and capsular genes.

Pattern Number	Code Number	Antimicrobial Resistance Pattern	MAR Index	Carbapenemase Genes	Capsular Genes
1	1 U	AMO, SXT, CXM, TPZ, FOX, CRO, FEB, CAZ, CTX, CIP, IMI, MEM, ERT	1.0	*bla_K_*_PC_	K1
2	3 U	AMO, SXT, CXM, TPZ, CRO, FEB, CAZ, CTX, CIP, IMI, MEM, ERT	0.92	*bla*_VIM_	K54
3	7 U	AMO, SXT, CXM, TPZ, FOX, CRO, FEB, CAZ, CIP, CTX, IMI, MEM, ERT	1.0	*bla_I_*_MP-1&2_	K1
4	9 U	AMO, SXT, CXM, TPZ, FOX, CRO, FEB, CAZ, CTX, IMI, MEM, ERT	0.92	*bla*_OXA-48_	K20
5	17 U	AMO, CXM, TPZ, FOX, CRO, FEB, CAZ, CTX, CIP, IMI, MEM, ERT	0.92	*bla*_VIM_	K1
6	19 U	AMO, SXT, CXM, FOX, CRO, FEB, CAZ, CTX, CIP, IMI, MEM, ERT	0.92	*bla*_OXA-48_	K54
7	23 U	AMO, SXT, CXM, TPZ, FOX, CRO, FEB, CAZ, CTX, IMI, MEM, ERT	0.92	*bla*_VIM_	K1
8	27 U	AMO, SXT, CXM, FOX, CRO, FEB, CAZ, CTX, IMI, MEM, ERT	0.85	*bla*_VIM_	K54
9	31 U	AMO, SXT, CXM, FOX, CRO, FEB, CAZ, CTX, IMI, MEM, ERT	0.85	*bla*_OXA-48_	K1
10	33 U	AMO, SXT, CXM, TPZ, FOX, CRO, FEB, CAZ, CTX, IMI, MEM, ERT	0.92	*bla*_VIM_	K20
11	43 U	AMO, SXT, CXM, FOX, CRO, FEB, CAZ, CTX, CIP, IMI, MEM, ERT	0.92	*bla*_VIM_	K57
12	45 U	AMO, SXT, CXM, FOX, CRO, FEB, CAZ, CTX, CIP, IMI, MEM, ERT	0.92	*bla*_IMP-1&2_	K54
13	48 U	AMO, SXT, CXM, TPZ, FOX, CRO, FEB, CAZ, CTX, CIP, IMI, MEM, ERT	1.0	*bla*_KPC_, *bla*_IMP-1&2_	K1
14	54 U	AMO, SXT, CXM, TPZ, FOX, CRO, FEB, CAZ, CTX, CIP, IMI, MEM, ERT	1.0	*bla*_VIM_	K57
15	58 U	AMO, SXT, CXM, TPZ, FOX, CRO, FEB, CAZ, CTX, IMI, MEM, ERT	0.92	*bla*_NDM_	K2
16	64 U	AMO, SXT, CXM, FOX, CRO, FEB, CAZ, CTX, CIP, IMI, MEM, ERT	0.92	*bla*_OXA-48_	K57
17	67 U	AMO, CXM, FOX, CRO, FEB, CAZ, CTX, IMI, MEM, ERT	0.77	*bla*_OXA-48_	K54
18	75 U	AMO, SXT, CXM, TPZ, FOX, CRO, FEB, CAZ, CTX, CIP, IMI, MEM, ERT	1.0	*bla*_VIM_	K54
19	77 U	AMO, SXT, CXM, FOX, CRO, FEB, CAZ, CTX, CIP, IMI, MEM, ERT	0.92	*bla*_VIM_	K1
20	79 U	AMO, SXT, CXM, TPZ, FOX, CRO, FEB, CAZ, CTX, CIP, IMI, MEM, ERT	1.0	*bla*_IMP-1&2,_ *bla*_OXA-48_	-
21	91 U	AMO, SXT, CXM, FOX, CRO, FEB, CAZ, CTX, CIP, IMI, MEM, ERT	0.92	*bla*_VIM_	K20
22	107 U	AMO, SXT, CXM, TPZ, FOX, CRO, FEB, CAZ, CTX, CIP	0.77	*bla*_VIM_, *bla*_OXA-48_	K1
23	110 U	AMO, SXT, CXM, TPZ, FOX, CRO, FEB, CAZ, CTX, CIP	0.77	*bla*_VIM_	K57
24	114 U	AMO, SXT, CXM, TPZ, FOX, CRO, FEB, CAZ, CTX, IMI, MEM, ERT	0.92	*bla*_VIM_	K1
25	116 U	AMO, SXT, CXM, FOX, CRO, FEB, CAZ, CTX, IMI, MEM, ERT	0.85	*bla*_VIM_, *bla*_OXA-48_	K54
26	121 U	AMO, SXT, CXM, TPZ, FOX, CRO, FEB, CAZ, CTX, CIP	0.77	*bla*_VIM_	K1
27	124 U	AMO, SXT, CXM, TPZ, FOX, CRO, FEB, CAZ, CTX, CIP	0.77	*bla*_OXA-48_	K1
28	128 U	AMO, SXT, CXM, FOX, CRO, FEB, CAZ, CTX, CIP, IMI, MEM, ERT	0.92	*bla*_KPC_	-
29	129 U	AMO, SXT, CXM, TPZ, FOX, CRO, FEB, CAZ, CTX, IMI, MEM, ERT	0.92	*bla*_NDM_, *bla*_KPC&,_*bla*_OXA-48_	K54
30	134 U	AMO, SXT, CXM, TPZ, FOX, CRO, FEB, CAZ, CTX, CIP	0.77	*bla*_OXA-48_	K20
31	137 U	AMO, SXT, CXM, TPZ, FOX, CRO, FEB, CAZ, CTX, CIP	0.77	*bla*_IMP-1&2_	-
32	139 U	AMO, SXT, CXM, TPZ, FOX, CRO, FEB, CAZ, CTX, CIP, IMI, ERT	0.92	*bla*_VIM_	K1
33	144 U	AMO, SXT, CXM, TPZ, FOX, CRO, FEB, CAZ, CTX, IMI, MEM, ERT	0.92	*bla*_VIM_	K1
34	156 U	AMO, SXT, CXM, TPZ, FOX, CRO, FEB, CAZ, CTX, CIP, IMI, MEM, ERT	1.0	*bla*_VIM_, *bla*_NDM_	K54
35	4 P	AMO, CXM, TPZ, FOX, CRO, FEB, CAZ, CTX, IMI, MEM, ERT	0.85	*bla*_OXA-48_	K57
36	15 P	AMO, CXM, FOX, CRO, FEB, CAZ, CTX, IMI, MEM, ERT	0.77	*bla*_NDM,_ *bla*_OXA-48_	K57
37	35 P	AMO, SXT, CXM, TPZ, CRO, FEB, CAZ, CTX, CTP, IMI, MEM, ERT	0.92	*bla*_KPC_	K57
38	42 P	AMO, SXT, CXM, TPZ, FOX, CRO, FEB, CAZ, CTX, CIP	0.77	*bla*_OXA-48_	K2
39	50 P	AMO, SXT, CXM, TPZ, FOX, CRO, FEB, CAZ, CTX, CIP, IMI, MEM, ERT	1.0	*bla*_OXA-48_	K20
40	66 P	AMO, SXT, CXM, FOX, CRO, FEB, CAZ, CTX, CIP	0.69	*bla*_VIM_	K2
41	69 P	AMO, SXT, CXM, TPZ, FOX, CRO, FEB, CAZ, CTX, CIP, IMI, MEM, ERT	1.0	*bla*_IMP-1&2_	K1
42	71 P	AMO, SXT, CXM, TPZ, FOX, CRO, FEB, CAZ, CTX, CIP	0.77	*bla*_OXA-48_	-
43	82 P	AMO, SXT, CXM, TPZ, FOX, CRO, FEB, CAZ, CTX, IMI, MEM, ERT	0.92	*bla*_KPC_	K57
44	87 P	AMO, SXT, CXM, TPZ, FOX, CRO, FEB, CAZ, CTX, IMI, MEM, ERT	0.92	*bla*_VIM_	-
45	89 P	AMO, SXT, CXM, FOX, CRO, FEB, CAZ, CTX, CIP, IMI, MEM, ERT	0.92	*bla*_OXA-48_	K57
46	96 P	AMO, SXT, CXM, TPZ, FOX, CRO, FEB, CAZ, CTX, CIP, IMI, MEM, ERT	1.0	*bla*_IMP-1&2,_ *bla*_OXA-48_	K1
47	98 P	AMO, SXT, CXM, TPZ, FOX, CRO, FEB, CAZ, CTX, IMI, MEM, ERT	0.92	*bla*_VIM_	-
48	106 P	AMO, SXT, CXM, TPZ, FOX, CRO FEB, CAZ, CTX, IMI, MEM, ERT	0.92	*bla*_IMP-1&2_	K54
49	113 P	AMO, SXT, CXM, TPZ, FOX, CRO, FEB, CAZ, CTX, CIP, IMI, ERT	0.92	*bla*_OXA-48_	K20
50	120 P	AMO, SXT, CXM, CRO, FEB, CAZ, CTX, CIP, IMI, MEM, ERT	0.85	*bla*_IMP-1&2_	K57
51	122 P	AMO, SXT, CXM, FOX, CRO, FEB, CAZ, CTX, CIP, IMI, MEM, ERT	0.92	*bla*_IMP-1&2_	K54
52	130 P	AMO, SXT, CXM, TPZ, FOX, CRO FEB, CAZ, CTX, CIP	0.77	*bla*_VIM_	K1
53	135 P	AMO, SXT, CXM, TPZ, FOX, CRO, FEB, CAZ, CTX, CTP, IMI, MEM, ERT	1.0	*bla*_IMP-1&2,_*bla*_NDM_, *bla*_OXA-48_	K57
54	140 P	AMO, SXT, CXM, TPZ, FOX, CRO, FEB, CAZ, CTX, CIP	0.77	*bla*_OXA-48_	K54
55	40 S	AMO, SXT, CXM, TPZ, CRO, FEB, CAZ, CTX, CTP, IMI, MEM, ERT	0.92	*bla*_NDM_	K1
56	62 S	AMO, SXT, CXM, TPZ, FOX, CRO, FEB, CAZ, CTX, CIP, IMI, MEM, ERT	1.0	*bla*_KPC_, *bla*_OXA-48_	K1
57	101 S	AMO, SXT, CXM, TPZ, FOX, CRO, FEB, CAZ, CTX, CIP	0.77	*bla*_OXA-48_	K57
58	151 S	AMO, SXT, CXM, FOX, CRO, FEB, CAZ, CTX, IMI, MEM, ERT	0.85	*bla*_IMP-1&2_	K57
59	60 B	AMO, SXT, CXM, TPZ, FOX, CRO, FEB, CAZ, CTX, IMI, MEM, ERT	0.92	*bla*_VIM_	K57
60	84 B	AMO, CXM, CRO, FEB, CAZ, CTX, IMI, MEM, ERT	0.69	*bla*_OXA-48_	K1
61	10 T	AMO, CXM, TPZ, FOX, CRO, FEB, CAZ, CTX, IMI, MEM, ERT	0.85	*bla*_VIM_	K2
62	24 T	AMO, SXT, CXM, TPZ, CRO, FEB, CAZ, CTX, IMI, MEM, ERT	0.85	*bla*_OXA-48_	K57

U: urine; P: pus; S: sputum; B: blood; T: tracheal aspirate; MAR: multiple antibiotic resistance; —: samples negative for tested capsular types; AMO: amoxicillin/clavulanic acid; CIP: ciprofloxacin; CXM: cefuroxime, TPZ: piperacillin tazobactam; FOX: cefoxitin; FEP: cefipime; CRO: ceftriaxone; CAZ: ceftazidime; CTX: cefotaxime; SXT: trimethoprim–sulfamethoxazole (SXT); IMI: imipenem (IMI); ERT: ertapenem; MEM: meropenem.

**Table 7:** Correlation between carbapenemases and capsular genes.

	*Bla*_OXA-48_ (n = 25)	*Bla*_VIM_ (n = 24)	*Bla*_IMP1&2_ (n = 12)	*bla*_KPC_ (n = 7)	*bla*_NDM_ (n = 6)	χ^2^	*p*-Value
K1	6 (24%)	9 (37.5%)	4 (33.3%)	3 (42.9%)	1 (16.7%)	2.170	0.733
K2	1 (4%)	2 (8.3%)	0 (0%)	0 (0%)	1 (16.7%)	2.876	0.481
K20	4 (16%)	2 (8.3%)	0 (0%)	0 (0%)	0 (0%)	2.663	0.554
K54	5 (20%)	5 (20.8%)	3 (25%)	1 (14.3%)	2 (33.3%)	1.152	0.932
K57	7 (28%)	4 (16.7%)	3 (25%)	2 (28.6%)	2 (33.3%)	1.752	0.817

χ^2^: Chi-square test.

## Data Availability

Data are accessible upon request from the corresponding author.

## References

[1] Ferri M., Ranucci E., Romagnoli P., Giaccone V. (2017). Antimicrobial resistance: A global emerging threat to public health systems. Crit. Rev. Food Sci. Nutr..

[2] European Food Safety Authority, European Centre for Disease Prevention and Control (2015). EU Summary Report on antimicrobial resistance in zoonotic and indicator bacteria from humans, animals and food in 2013. EFSA J..

[3] Bassetti M., Righi E., Carnelutti A., Graziano E., Russo A. (2018). Multidrug-resistant *Klebsiella pneumoniae*: Challenges for treatment, prevention and infection control. Expert Rev. Anti-Infect. Ther..

[4] Pendleton J.N., Gormanm S.P., Gilmore B.F. (2013). Clinical relevance of the ESKAPE pathogens. Expert Rev. Anti-Infect Ther..

[5] Bengoechea J.A., Sa Pessoa J. (2019). *Klebsiella pneumoniae* infection biology: Living to counteract host defences. FEMS Microbiol. Rev..

[6] Chen L., Kreiswirth B.N. (2018). Convergence of carbapenem-resistance and hypervirulence in *Klebsiella pneumoniae*. Lancet Infect. Dis..

[7] Wyres K., Holt K.E. (2018). *Klebsiella pneumoniae* as a key trafficker of drug resistance genes from environmental to clinically important bacteria. Curr. Opin. Microbiol..

[8] Kang Y., Tian P., Tan T. (2015). Research advances in the virulence factors of Klebsiella pneumonia—A review. Acta Microbiol. Sin.

[9] Cortés G., Borrell N., de Astorza B., Gómez C., Sauleda J., Albertí S. (2002). Molecular analysis of the contribution of the capsular polysaccharide and the lipopolysaccharide O side chain to the virulence of *Klebsiella pneumoniae* in a murine model of pneumonia. Infect. Immun..

[10] Siri G.P., Sithebe N.P., Ateba C.N. (2011). Identification of *Klebsiella* species isolate from Modimola dam (Mafikeng) North West Province South Africa. J. Afr. J. Microbiol. Res..

[11] Fevre C., Passet V., Deletoile A., Barbe V., Frangeul L., Almeida A.S., Brisse S. (2011). PCR-based identification of *Klebsiella pneumoniae* subsp. rhinoscleromatis, the agent of rhinoscleroma. PLoS Negl. Trop. Dis..

[12] Wyres K.L., Wick R.R., Gorrie C., Jenney A., Follador R., Thomson N.R., Holt K.E. (2016). Identification of Klebsiella capsule synthesis loci from whole genome data. Microb. Genom..

[13] Pan Y.J., Lin T.L., Chen C.T., Chen Y.Y., Hsieh P.F., Hsu C.R., Wang J.T. (2015). Genetic analysis of capsular polysaccharide synthesis gene clusters in 79 capsular types of *Klebsiella* spp. Sci. Rep..

[14] Siu L.K., Fung C.P., Chang F.Y., Lee N., Yeh K.M., Koh T.H., Ip M. (2011). Molecular typing and virulence analysis of serotype K1 *Klebsiella pneumoniae* strains isolated from liver abscess patients and stool samples from noninfectious subjects in Hong Kong, Singapore, and Taiwan. J. Clin. Microbiol..

[15] Cheng L., Cao X.L., Shen H., Zhang Z.F., Ning M.Z., Zhou W.Q. (2015). Investigations on the virulence, serotypes and genotyping of *Klebsiella pneumoniae* producing KPC-2. Chin. J. Clin. Lab. Sci..

[16] Wasfi R., Elkhatib F.W., Ashour M.H. (2016). Molecular typing and virulence analysis of multidrug resistant *Klebsiella pneumoniae* clinical isolates recovered from Egyptian hospitals. Sci. Rep..

[17] Mohamed E.R., Ali M.Y., Waly N.G.F.M., Halby H.M., El-Baky R.M.A. (2019). The Inc FII Plasmid and its Contribution in the Transmission of blaNDM-1 and blaKPC-2 in *Klebsiella pneumoniae* in Egypt. Antibiotics.

[18] Forbes B.A., Sahm D.F., Weissfeld A.S. (2016). Study Guide for Bailey and Scott’s Diagnostic Microbiology-E-Book.

[19] Clinical and Laboratory Standards Institute [CLSI] (2021). Performance Standards for Antimicrobial Susceptibility Testing.

[20] Tambekar D., Dhanorkar D., Gulhane S., Khandelwal V., Dudhane M. (2006). Antibacterial susceptibility of some urinary tract pathogens to commonly used antibiotics. Afr. J. Biotechnol..

[21] Zhang S., Yang G., Ye Q., Wu Q., Zhang J., Huang Y. (2018). Phenotypic and Genotypic Characterization of *Klebsiella pneumoniae* Isolated From Retail Foods in China. Front. Microbiol..

[22] Dallenne C., Costa A.D., Decré D., Favier C., Arlet G. (2010). Development of a set of multiplex PCR assays for the detection of genes encoding important β-lactamases in *Enterobacteriaceae*. J. Antimicrob. Chemother..

[23] Ssekatawa K., Byarugaba D.K., Nakavuma J.L., Kato C.D., Ejobi F., Tweyongyere R., Eddie W.M. (2021). Prevalence of pathogenic *Klebsiella pneumoniae* based on PCR capsular typing harbouring carbapenemases encoding genes in Uganda tertiary hospitals. Antimicrob. Resist. Infect. Control.

[24] Holt K.E., Wertheim H., Zadoks R.N., Baker S., Whitehouse C.A., Dance D., Thomson N.R. (2015). Genomic analysis of diversity, population structure, virulence, and antimicrobial resistance in *Klebsiella pneumoniae*, an urgent threat to public health. Proc. Natl. Acad. Sci. USA.

[25] Twenhafel N.A., Whitehouse C.A., Stevens E.L., Hottel H.E., Foster C.D., Gamble S., Steele K.E. (2008). Multisystemic abscesses in African green monkeys (Chlorocebus aethiops) with invasive *Klebsiella pneumoniae*—Identification of the hypermucoviscosity phenotype. Vet. Pathol..

[26] Gaiarsa S., Comandatore F., Gaibani P., Corbella M., Dalla Valle C., Epis S., Sassera D. (2015). Genomic epidemiology of *Klebsiella pneumoniae* in Italy and novel insights into the origin and global evolution of its resistance to carbapenem antibiotics. Antimicrob. Agents Chemother..

[27] Abo Samra M.A.A., Ali N.K., El-Madboly A.A.E. (2019). Detection of Multi-Drug Resistant *Klebsiella pneumoniae* in Al-Zahraa University Hospital. Egypt. J. Hosp. Med..

[28] Parrott A.M., Shi J., Aaron J., Green D.A., Whittier S., Wu F. (2021). Detection of multiple hypervirulent *Klebsiella pneumoniae* strains in a New York City hospital through screening of virulence genes. Clin. Microbiol. Infect..

[29] Palmeiro J.K., De Souza R.F., Schörner M.A., Passarelli-Araujo H., Grazziotin A.L., Vidal N.M., Dalla-Costa L.M. (2019). Molecular epidemiology of multidrug-resistant *Klebsiella pneumoniae* isolates in a Brazilian tertiary hospital. Front. Microbiol..

[30] Sedighi P., Zarei O., Karimi K., Taheri M., Karami P., Shokoohizadeh L. (2020). Molecular typing of *Klebsiella pneumoniae* clinical isolates by Enterobacterial repetitive intergenic consensus polymerase chain reaction. Int. J. Microbiol..

[31] Farhadi M., Ahanjan M., Goli H.R., Haghshenas M.R., Gholami M. (2021). High frequency of multidrug-resistant (MDR) *Klebsiella pneumoniae* harboring several β-lactamase and integron genes collected from several hospitals in the north of Iran. Ann. Clin. Microbiol. Antimicrob..

[32] Pereira A., Petrucci T., Simões M.J. (2017). *Klebsiella pneumoniae* do Serotipo K1 e do Clone Hipervirulento ST23: Primeiro Caso Documentado em Portuga. Acta Med. Port..

[33] Moghadas A.J., Kalantari F., Sarfi M., Shahhoseini S., Mirkalantari S. (2018). Evaluation of virulence factors and antibiotic resistance patterns in clinical urine isolates of *Klebsiella pneumoniae* in Semnan, Iran. Jundishapur J. Microbiol..

[34] Kotb S., Lyman M., Ismail G., Abd El Fattah M., Girgis S.A., Etman A., Talaat M. (2020). Epidemiology of carbapenem-resistant *Enterobacteriaceae* in Egyptian intensive care units using National Healthcare–associated Infections Surveillance Data, 2011–2017. Antimicrob. Resist. Infect. Control.

[35] ElMahallawy H.A., Zafer M.M., Amin M.A., Ragab M.M., Al-Agamy M.H. (2018). Spread of carbapenem resistant *Enterobacteriaceae* at tertiary care cancer hospital in Egypt. Infect. Dis..

[36] Ogbolu D.O., Webber M.A. (2014). High-level and novel mechanisms of carbapenem resistance in Gram-negative bacteria from tertiary hospitals in Nigeria. Int. J. Antimicrob. Agents.

[37] Elramalli A., Almshawt N., Ahmed M.O. (2017). Current problematic and emergence of carbapenemase-producing bacteria: A brief report from a Libyan hospital. Pan Afr. Med. J..

[38] Tsai Y.M., Wang S., Chiu H.C., Kao C.Y., Wen L.L. (2020). Combination of modified carbapenem inactivation method (mCIM) and EDTA-CIM (eCIM) for phenotypic detection of carbapenemase-producing *Enterobacteriaceae*. BMC Microbiol..

[39] Raheel A., Azab H., Hessam W., Abbadi S., Ezzat A. (2020). Detection of carbapenemase enzymes and genes among carbapenem-resistant *Enterobacteriaceae* isolates in Suez Canal University Hospitals in Ismailia, Egypt. Microbes Infect. Dis..

[40] Perovic O., Ismail H., Quan V., Bamford C., Nana T., Chibabhai V., Singh-Moodley A. (2020). Carbapenem-resistant *Enterobacteriaceae* in patients with bacteraemia at tertiary hospitals in South Africa, 2015 to 2018. Eur. J. Clin. Microbiol. Infect. Dis..

[41] Kollenda H., Frickmann H., Helal R.B., Wiemer D.F., Naija H., El Asli M.S., Moussa M.B. (2019). Screening for carbapenemases in ertapenem-resistant *Enterobacteriaceae* collected at a Tunisian hospital between 2014 and 2018. Eur. J. Microbiol. Immunol..

[42] Mahrach Y., Mourabit N., Arakrak A., Bakkali M., Laglaoui A. (2019). Phenotypic and molecular study of carbapenemase-producing *Enterobacteriaceae* in a regional hospital in northern Morocco. J. Clin. Med. Sci..

[43] Nordmann P., Naas T., Poirel L. (2011). Global spread of carbapenemase-producing *Enterobacteriaceae*. Emerg. Infect. Dis..

[44] Lopes E., Saavedra M.J., Costa E., de Lencastre H., Poirel L., Aires-de-Sousa M. (2020). Epidemiology of carbapenemase-producing *Klebsiella pneumoniae* in northern Portugal: Predominance of KPC-2 and OXA-48. J. Glob. Antimicrob. Resist..

[45] Hussein N.H., Hussein AL-Kakei S.N., Taha B.M. (2022). The predominance of *Klebsiella pneumoniae* carbapenemase (KPC-type) gene among high-level carbapenem-resistant *Klebsiella pneumoniae* isolates in Baghdad, Iraq. Mol. Biol. Rep..

[46] Elmonir W., Abd El-Aziz N.K., Tartor Y.H., Moustafa S.M., Abo Remela E.M., Eissa R., Saad H.A., Tawab A.A. (2021). Emergence of Colistin and Carbapenem Resistance in Extended-Spectrum β-Lactamase Producing *Klebsiella pneumoniae* Isolated from Chickens and Humans in Egypt. Biology.

[47] Singh-Moodley A., Perovic O. (2016). Antimicrobial susceptibility testing in predicting the presence of carbapenemase genes in *Enterobacteriaceae* in South Africa. BMC Infect. Dis..

[48] Kateete D.P., Nakanjako R., Namugenyi J., Erume J., Joloba M.L., Najjuka C.F. (2016). Carbapenem resistant *Pseudomonas aeruginosa* and *Acinetobacter baumannii* at Mulago hospital in Kampala, Uganda (2007–2009). Springerplus.

[49] Okoche D., Asiimwe B.B., Katabazi F.A., Kato L., Najjuka C.F. (2015). Prevalence and characterization of carbapenem-resistant *Enterobacteriaceae* isolated from Mulago National Referral Hospital, Uganda. PLoS ONE.

[50] Ampaire L.M., Katawera V., Nyehangane D., Boum Y., Bazira J. (2015). Epidemiology of carbapenem resistance among multi-drug resistant *Enterobacteriaceae* in Uganda. Br. Microbiol. Res. J..

[51] Wade D.M., Hankins M., Smyth D.A., Rhone E.E., Mythen M.G., Howell D.C., Weinman J.A. (2014). Detecting acute distress and risk of future psychological morbidity in critically ill patients: Validation of the intensive care psychological assessment tool. Crit. Care.

[52] Masseron A., Poirel L., Ali B.J., Syed M.A., Nordmann P. (2019). Molecular characterization of multidrug-resistance in Gram-negative bacteria from the Peshawar teaching hospital, Pakistan. New Microbes New Infect..

[53] Sadeghi M.R., Ghotaslou R., Akhi M.T., Asgharzadeh M., Hasani A. (2016). Molecular characterization of extended-spectrum β-lactamase, plasmid-mediated AmpC cephalosporinase and carbapenemase genes among *Enterobacteriaceae* isolates in five medical centres of East and West Azerbaijan, Iran. J. Med. Microbiol..

[54] Haji S.H., Aka S.T.H., Ali F.A. (2021). Prevalence and characterisation of carbapenemase encoding genes in multidrug-resistant Gram-negative bacilli. PLoS ONE.

[55] Solgi H., Badmasti F., Aminzadeh Z., Giske C.G., Pourahmad M., Vaziri F., Shahcheraghi F. (2017). Molecular characterization of intestinal carriage of carbapenem-resistant *Enterobacteriaceae* among inpatients at two Iranian university hospitals: First report of co-production of bla NDM-7 and bla OXA-48. Eur. J. Clin. Microbiol. Infect Dis..

[56] Di Tella D., Tamburro M., Guerrizio G., Fanelli I., Sammarco M.L., Ripabelli G. (2019). Molecular Epidemiological Insights into Colistin-Resistant and Carbapenemases-Producing Clinical *Klebsiella pneumoniae* Isolates. Infect Drug Resist..

[57] Nordmann P., Dortet L., Poirel L. (2012). Carbapenem resistance in *Enterobacteriaceae*: Here is the storm!. J. Trends Mol. Med..

[58] Codjoe F.S., Donkor E.S. (2017). Carbapenem resistance: A review. Med. Sci..

[59] Fang C.T., Lai S.Y., Yi W.C., Hsueh P.R., Liu K.L., Chang S.C. (2007). *Klebsiella pneumoniae* genotype K1: An emerging pathogen that causes septic ocular or central nervous system complications from pyogenic liver abscess. Clin. Infect Dis..

[60] Fung C.P., Chang F.Y., Lee S.C., Hu B.S., Kuo B.I., Liu C.Y., Siu L.K. (2002). A global emerging disease of *Klebsiella pneumoniae* liver abscess: Is serotype K1 an important factor for complicated endophthalmitis?. Gut.

[61] Pan Y.J., Fang H.C., Yang H.C., Lin T.L., Hsieh P.F., Tsai F.C., Wang J.T. (2008). Capsular polysaccharide synthesis regions in *Klebsiella pneumoniae* serotype K57 and a new capsular serotype. J. Clin. Microbiol..

[62] Lin Y.T., Wang Y.P., Wang F.D., Fung C.P. (2015). Community-onset *Klebsiella pneumoniae* pneumonia in Taiwan: Clinical featurof the disease and associated microbiological characteristics of isolates from pneumonia and nasopharynx. Front. Microbiol..

[63] Chuang Y.P., Fang C.T., Lai S.Y., Chang S.C., Wang J.T. (2006). Genetic determinants of capsular serotype K1 of *Klebsiella pneumoniae* causing primary pyogenic liver abscess. J. Infect Dis..

[64] Paczosa M.K., Mecsas J. (2016). *Klebsiella pneumoniae*: Going on the offense with a strong defense. Microbiol. Mol. Biol. Rev..

[65] Choi M., Hegerle N., Nkeze J., Sen S., Jamindar S., Nasrin S., Sen S., Permala-Booth J., Sinclair J., Tapia M.D. (2020). The Diversity of Lipopolysaccharide (O) and Capsular Polysaccharide (K) Antigens of Invasive *Klebsiella pneumoniae* in a Multi-Country Collection. Front. Microbiol..

[66] Soltani E., Hasani A., Rezaee M.A., Nahandi M., Hasani A., Gholizadeh P. (2022). An Alliance of Carbapenem-Resistant *Klebsiella pneumoniae* with Precise Capsular Serotypes and Clinical Determinants: A Disquietude in Hospital Setting. Can. J. Infect Dis. Med. Microbiol..

